# Epidemiology and antimicrobial resistance of invasive non-typhoidal Salmonellosis in rural Thailand from 2006-2014

**DOI:** 10.1371/journal.pntd.0006718

**Published:** 2018-08-06

**Authors:** Toni Whistler, Patranuch Sapchookul, David W. McCormick, Ornuma Sangwichian, Possawat Jorakate, Sirirat Makprasert, Anchalee Jatapai, Sathapana Naorat, Uraiwan Surin, Surathinee Koosakunwat, Surachai Supcharassaeng, Barameht Piralam, Mathew Mikoleit, Henry C. Baggett, Julia Rhodes, Christopher J. Gregory

**Affiliations:** 1 Thailand Ministry of Public Health—US Centers for Disease Control and Prevention Collaboration (TUC), Nonthaburi, Thailand; 2 Division of Global Health Protection, Centers for Disease Control and Prevention, Atlanta, Georgia, United States of America; 3 Nakhon Phanom General Hospital, Nakhon Phanom Provincial Health Office, Nakhon Phanom, Thailand; 4 Sa Kaeo Crown Prince Hospital, Sa Kaeo Provincial Health Office, Ministry of Public Health, Thailand; Oxford University Clinical Research Unit Vietnam, VIET NAM

## Abstract

**Introduction:**

Invasive salmonellosis is a common cause of bloodstream infection in Southeast Asia. Limited epidemiologic and antimicrobial resistance data are available from the region.

**Methods:**

Blood cultures performed in all 20 hospitals in the northeastern province of Nakhon Phanom (NP) and eastern province of Sa Kaeo (SK), Thailand were captured in a bloodstream infection surveillance system. Cultures were performed as clinically indicated in hospitalized patients; patients with multiple positive cultures had only the first included. Bottles were incubated using the BacT/Alert system (bioMérieux, Thailand) and isolates were identified using standard microbiological techniques; all *Salmonella* isolates were classified to at least the serogroup level. Antimicrobial resistance was assessed using disk diffusion.

**Results:**

*Salmonella* was the fifth most common pathogen identified in 147,535 cultures with 525 cases (211 in Nakhon Phanom (NP) and 314 in Sa Kaeo (SK)). The overall adjusted iNTS incidence rate in NP was 4.0 cases/100,000 person-years (95% CI 3.5–4.5) and in SK 6.4 cases/100,000 person-years (95% CI 5.7–7.1; p = 0.001). The most common serogroups were C (39.4%), D (35.0%) and B (9.9%). Serogroup D predominated in NP (103/211) with 59.2% of this serogroup being *Salmonella* serovar Enteritidis. Serogroup C predominated in SK (166/314) with 84.3% of this serogroup being *Salmonella* serovar Choleraesuis. Antibiotic resistance was 68.2% (343/503) for ampicillin, 1.2% (6/482) for ciprofloxacin (or 58.1% (280/482) if both intermediate and resistant phenotypes are considered), 17.0% (87/512) for trimethoprim-sulfamethoxazole, and 12.2% (59/484) for third-generation cephalosporins (cefotaxime or ceftazidime). Multidrug resistance was seen in 99/516 isolates (19.2%).

**Conclusions:**

The NTS isolates causing bloodstream infections in rural Thailand are commonly resistant to ampicillin, cefotaxime, and TMP-SMX. Observed differences between NP and SK indicate that serogroup distribution and antibiotic resistance may substantially differ throughout Thailand and the region.

## Author summary

Invasive strains of non-typhoidal salmonella (iNTS) are a common cause of bloodstream infection in Southeast Asia, however limited epidemiologic and antimicrobial resistance data are available and no population-based studies have been published from the region. We use nine years of bloodstream infections data from surveillance conducted in two Thai border provinces to report on the incidence, serotype distribution and antimicrobial resistance patterns. Incidence of iNTS was 5.1 cases/100,000 population (95% CI 4.7–5.6), likely an under estimate of the true burden of disease as we only used hospitalized cases and less sensitive blood culture for bacterial isolation. Levels of drug non-susceptibility were high among isolates, especially for fluoroquinolones such as ciprofloxacin (~60%) and third-generation cephalosporins such as cefotaxime or ceftazidime (~15%) that are commonly prescribed to provide empirical coverage against these pathogens. Multidrug resistance was widespread at 20% of isolates. Our data emphasizes the need for increased microbiological surveillance, and support the need for rational antibiotic therapy and the development of treatment guidelines.

## Introduction

Invasive non-typhoidal *Salmonella* (iNTS) disease, frequently associated with extremes of age, clinical malaria, HIV infections and malnutrition can be fatal in up to 20–25% of patients [1, 2]. The vast majority of reported iNTS infections and related deaths occur in Africa [3], where iNTS antimicrobial resistance against first line antibiotics chloramphenicol, ampicillin and trimethoprim/sulfamethoxazole (TMP/SMX) is high [3, 4]. Resistance to third-generation cephalosporins and fluoroquinolones has also been reported in several African countries [5, 6]. In contrast, the epidemiology and clinical manifestations of iNTS disease in Asia are not well documented. Compared to Africa where incidence of iNTS has been shown to vary over 100 fold based on location and age-group [2, 7], no population based estimates of iNTS are currently available from Asia. A 2017 review on iNTS global distribution showed a low number of reported cases in South and Southeast Asia [8] accompanied by a poor understanding of antimicrobial susceptibility patterns.

Disease surveillance is important in understanding temporal changes of *Salmonella* serovar isolates and variations in their antimicrobial susceptibility profiles in urban and rural settings. These data are essential to improving clinical care, updating treatment guidelines, and guiding public health interventions. To better understand the burden and antimicrobial resistance of iNTS disease in Thailand we analyzed data from population-based bloodstream infection surveillance in two rural Thai provinces from January 2006 through December 2014.

## Methods

### Ethical statement

The CDC Human Subjects Review Office reviewed this protocol and determined this study to be a routine public health activity not involving human subject research (CGH Determination and Approval number 2014–273). Surveillance is considered a core function of the Thai Ministry of Public Health and as such was determined not to require ethical committee or institutional review board evaluation. All data were anonymized prior to analysis.

### Study setting and population

**Fig 1:**
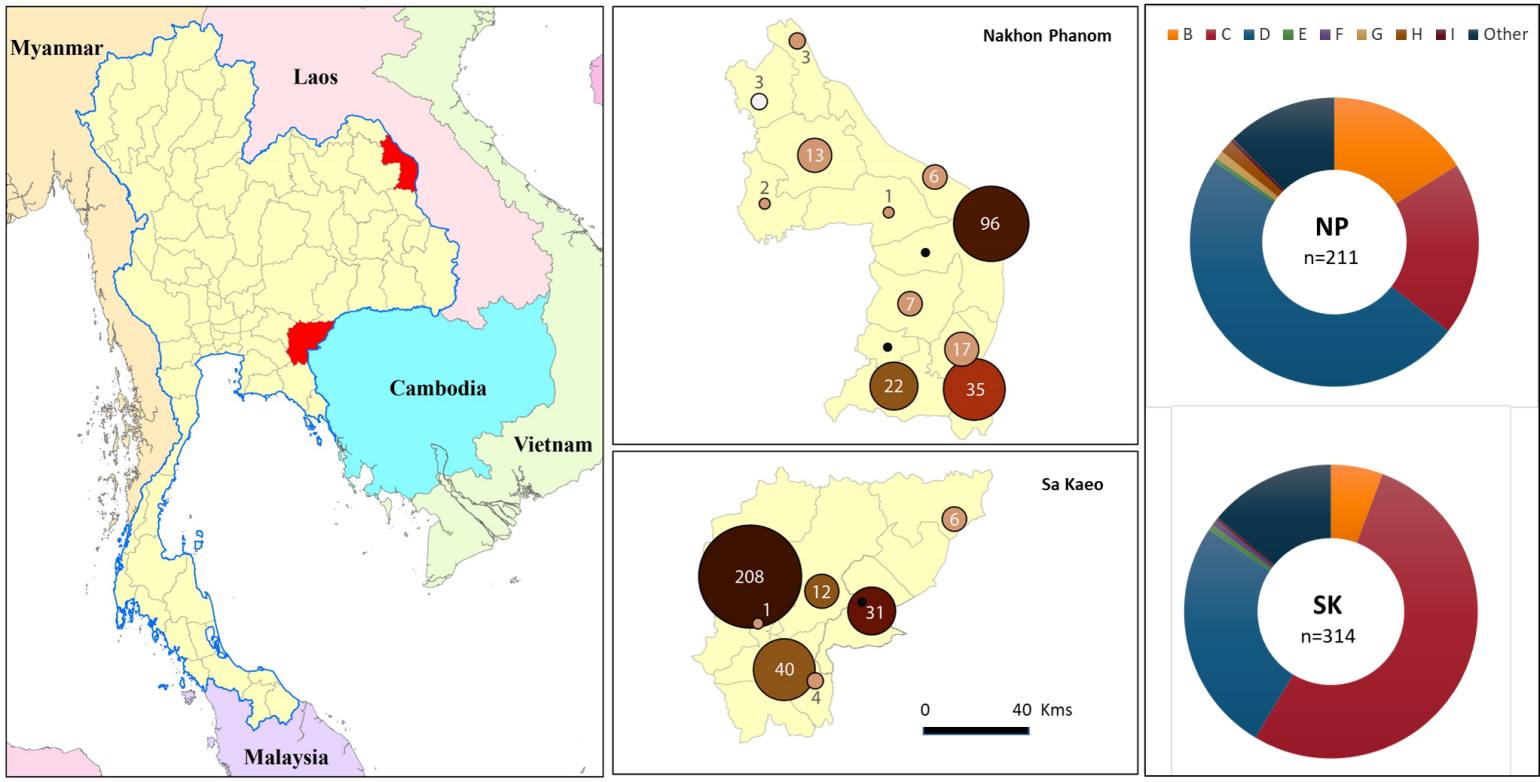
Map of Thailand showing the two rural provinces, Nakhon Phanom (NP) and Sa Kaeo (SK), participating in population-based bloodstream surveillance. The enlarged map of each province details the number of invasive non-typhoidal Salmonella (iNTS) cases for each hospital as indicated by the size of circle in each district. The color of the marker represents the number of beds for each hospital (the darker the color the higher the number of beds: range from 10–306). Map generated using ArcGIS version 10.5.1, (Environmental Systems Research Institute, Redlands, CA, USA); the base-layer country map was from the ArcGIS software and provincial level overlay file was obtained from the Ministry of Land, Thailand. Donut diagrams show the distribution of iNTS serogroups isolated in each province from 2006 to 2014.

This is a retrospective analysis of population-based surveillance data collected from January 2006 through December 2014 from two rural provinces in Thailand. Nakhon Phanom (NP), located in northeastern Thailand near the Lao People’s Democratic Republic border, and Sa Kaeo (SK), situated in eastern Thailand bordering Cambodia. Surveillance covered all hospitals in these provinces, twelve from NP and eight from SK, including two provincial hospitals (225–327 beds first-line, referral hospital), sixteen district hospitals, and two military hospitals (10–140 beds serving local communities) (Fig 1). Initially sites were selected for their ability to capture all potential admissions in the provinces, as there are no private or other acute care hospitals in either province. Starting in 2010, a subset of cases were also captured as part of an Invasive Bacterial Infection Surveillance (IBIS) system which provided additional clinical and laboratory testing information.

Both provinces are predominantly rural with agriculture being the major industry. Population estimates from National Economic and Social Development Board (NESDB) of Thailand projections based on 2010 census data estimated the population of Sa Kaeo at approximately 526,000 and NP at 734,000 [9].

### Specimen collection and laboratory testing

Cases were enrolled using the Thai Sepsis Guidelines (2006; updated 2010) based on Systemic Inflammatory Response Syndrome (SIRS). Sepsis is defined as having suspected or confirmed infection with at least two of the following SIRS criteria: temperature >38°C or <36°C, heart rate > 90 beats/min, respiratory rate >20/min or PaCO_2_ <32 mmHg, white blood cell count >12,000 or <4,000/mm^3^ or having band form neutrophils >10%.

Blood culture was performed as clinically indicated. Study nurses collected blood from hospitalized patients with every attempt made to do this prior to antibiotic administration. Blood was inoculated into an aerobic bottle (target volumes of 10 ml into a BacT/ALERT FA for patients aged ≥ 5 years, and 4 mL into a BacT/ALERT PF for children under 5 years). If more blood was available a BacT/ALERT MB bottle (target volume 3 ml) for enhanced growth for mycobacteria and other fastidious organisms was inoculated. In October 2011 the protocol was modified so MB bottles were available only by physician request and not routinely processed [10].

After collection at the district hospitals, blood culture bottles were sent to the provincial hospital for processing within 24 hours of collection, using a temperature controlled container (25–30⁰C) with continuous temperature monitoring. Upon arrival, cultures were incubated using an automated blood culture system (BacT/ALERT 3D, bioMérieux, U.S.A.) and monitored for growth at 35°C for up to 5 (FA/PF bottles) and 42 days (MB bottles), or until the instrument signaled a positive result for growth (i.e., alarm positive). Media from alarm positive bottles was sub-cultured onto sheep blood, chocolate and MacConkey agar plates and incubated overnight at 35°C, when standard biochemical testing was used for identification (Jorgensen, et al. 2015). Confirmatory pathogen identification was performed at Thailand’s National Institute of Health through the end of 2010. The provincial laboratories participated in national external quality assurance (EQA) programs throughout the surveillance period and an international EQA program (Royal College of Pathologists of Australia Quality Assurance Programs Pty. Ltd., Australia) was implemented in 2011 and continued throughout the remainder of the surveillance period.

The following organisms were considered likely contaminants and not included in the analysis: S*treptococcus viridans* serogroup, *Corynebacterium* spp., *Bacillus* spp. (other than *B*. *anthracis*), *Staphylococcus* spp. (other than *S*. *aureus*), and *Aerococcus* spp. All other bacteria were considered as clinically significant organisms. iNTS was considered to be present if either culture bottle (FA/PF or MB) grew non-typhoidal *Salmonella* species, even if a contaminant grew in the same bottle. For patients with multiple positive cultures, the first pathogen positive culture was included as a case. Repeat positive cultures that grew the same species within 30 days were excluded.

Antimicrobial susceptibility testing (AST) was performed using the disk diffusion method throughout the study period. Provincial authorities, taking into account local considerations such as available antibiotics and observed resistance patterns, guided antibiotics used for testing. Zone information for this manuscript was interpreted according to Clinical and Laboratory Standards Institute (CLSI) guidelines 2015 [11]. Consequently, testing for specific antibiotics was not uniform between the two provinces. All isolates were tested for resistance to ampicillin, TMP/SMX, cefotaxime or ceftazidime and ciprofloxacin. *Escherichia coli* ATCC 25922 was used as a reference standard. Intermediately resistant strains were included in the resistant category. Multidrug resistance (MDR) was defined as non-susceptibility (intermediate and resistant) to ≥3 antibiotics.

The typing methodologies applied to our *Salmonella* isolates varied over time. A total of 28 isolates (5.3%) were not characterized beyond their biochemical profile; these are recorded as *Salmonella enterica*. Many isolates, 308/525 (58.7%) were typed through the IBIS sentinel system at the Thailand National Institute of Health, where both O and H antigens were determined; whereas iNTS isolates from population-based surveillance were typed using O-group antigen sera, (A through I only; S & A Reagents Laboratory, Ltd, Bangkok, Thailand). Where cases were part of both surveillance systems, results from the former system were used. Serotypes were assigned according to the Kauffmann-White scheme [12, 13].

Glycerol stocks were prepared for -70°C long-term storage of all isolates.

### Statistical/Data analysis

Demographic and clinical data was collected from patients as part of ongoing public health surveillance activities. Data quality control was maintained through clearly defined data elements, data review at both collection and entry and system-based controls in the computerized data management system.

Age-stratified population estimates for the two provinces from 2010–2014 were obtained from the 2010 National Economic and Social Development Board (NESDB) census data. For the period 2007–2009, the 2010 NESDB age distribution was applied to the 2007–2009 NESDB overall provincial population estimates [14], as official intercensal estimates were not available.

Categorical variables were compared with Chi^2^ and Fisher’s exact test and continuous variables by the Student’s t test, ANOVA, Mann- Whitney U, and Kruskal-Wallis tests as appropriate. All statistical analyses were performed using Stata version 11 (StataCorp, College Station, TX, USA).

Ninety-five percent confidence intervals (CIs) on incidence estimates were calculated based on a Poisson distribution using the exact method. Trends over time in incidence rates were calculated by fitting a regression line to annual point estimates.

## Results

**Fig 2:**
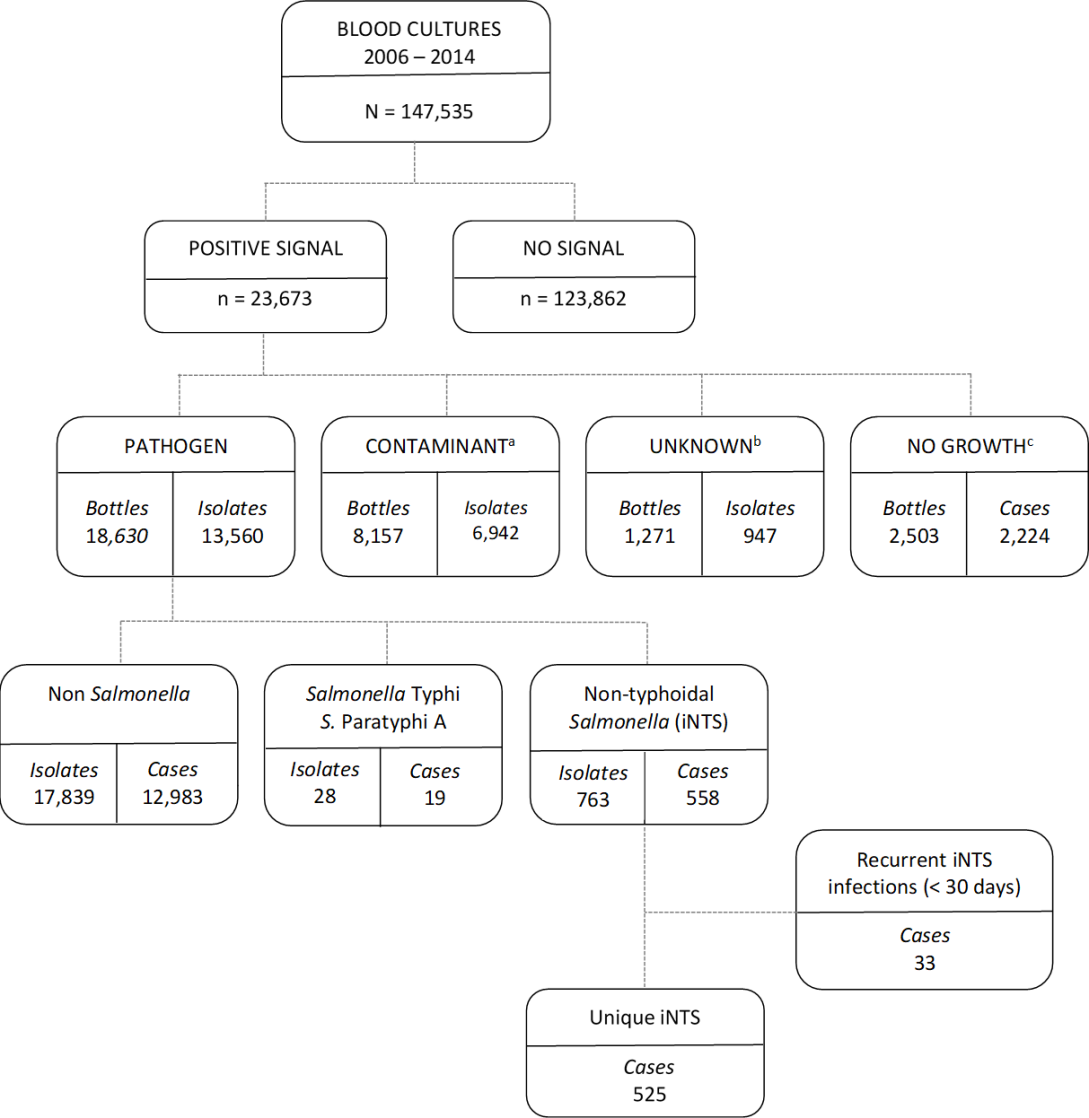
Flow diagram of blood culture data identifying non-typhoidal Salmonella cases, Nakhon Phanom and Sa Kaeo provinces, 2006-2014. Blood culture bottles were incubated at 35°C for up to 5 and 42 days (F and MB bottles respectively), or until the instrument signaled positive for growth (i.e., positive signal). Culture bottles that did not signal positive after the specified incubation periods (no signal) were considered to be negative without terminal subculture. A. The following organisms were considered likely contaminants and not included in the analysis: S*treptococcus viridans* group, *Corynebacterium* spp., *Bacillus* spp. (other than *B*. *anthracis*), *Staphylococcus* spp. (other than *S*. *aureus*), and *Aerococcus* spp. B. The unknown category were non-identifiable isolates using our standard biochemical testing including Gram-positive and negative cocci (n = 197 and 11 respectively), and Gram-positive bacilli (n = 343); these are likely contaminants. Gram-negative bacilli (n = 12) and unknown other (n = 708). C. The automated blood culture instrument signaled positive, but subculture yielded no organism.

Of 147,535 blood cultures plated from 2006–2014, 23,673 bottles were alarm positive (16.0%), and 13,560 clinically significant organisms were identified (8.6%). Among these pathogens, 791 isolates were *Salmonella* spp., of which 763 were iNTS, representing 558 separate cases (Fig 2). Target processing times of placement on instrument within 24 hours of collection were achieved 95.0% of the time (725/763) of the iNTS isolate bottles with a mean time to instrument placement of 7.5 hours (SD ± 7.7 hours). For the 38 bottles placed after 24 hours the mean time to placement was 39.8 hours (SD ± 35.6 hours). Isolation from an FA bottle only occurred 191 times, 60 isolations in the PF bottle alone and 2 only in MB bottles; 490 and 20 isolations were made in both a FA and MB or PF and MB bottle respectively. Thirty-three cases represented a second or third isolation of *Salmonella* within 30 days (31 people, 11 from Nakhon Phanom and 20 from Sa Kaeo) (Fig 2); the final unique case count for our analyses was 525. During this period only 19 cases of typhoid were identified, which included 12 cases of *Salmonella* serovar Typhi (5 cases in NP and 7 in SK) and 7 cases of *Salmonella* serovar Paratyphi A (6 cases in NP and 1 in SK). The proportion of iNTS cases to total number of pathogenic isolates made for NP and SK were 2.9% (211/7387) and 5.1% (314/6173) respectively. Only 2.5% of iNTS cases (13/525) had an additional pathogen identified in their bloodstream: 4 *Streptococcus* species (one of which was *S*. *pneumoniae*), 2 each of *Escherichia coli*, *Klebsiella pneumoniae* and *Pseudomonas aeruginosa*, and 1 each of *Listeria monocytogenes*, *Proteus* spp. and *Enterococcus* spp.

Patient demographics and clinical characteristics are summarized in Table 1. The age of patients with iNTS ranged from 2 months to 92 years, with a median age of 45 years. Overall, the highest number of cases occurred in patients between 35–49 years of age (145/525, 27.6%; Table 1). Children less than 5 years of age represented 11.6% of cases (61/525), with 11/16 (68.8%) of the cases in NP being in infants <1 year old, compared to 20/45 (44.4%) in SK (Table 1), no cases were seen in infants under 2 months of age.

**Table 1:** Demographic and clinical characteristics of patients with invasive non-typhoidal Salmonellosis in Nakhon Phanom and Sa Kaeo provinces, 2006–2014.

	Nakhon Phanom (n = 211) n (%)	Sa Kaeo (n = 314) n (%)	*p*-value
**Age in years**			0.001
< 5	16 (7.6)	45 (14.3)	0.115
5–19	11 (5.2)	17 (5.4)	1
20–34	34 (16.1)	53 (16.9)	Ref
35–49	45 (21.3)	100 (31.8)	0.252
50–64	55 (26.1)	46 (14.6)	0.041
> 65	50 (23.7)	53 (16.9)	0.241
**Sex (n = 248)**			0.601
Male	61 (61.0)	85 (57.4)	
Female	39 (39.0)	63 (42.6)	
**Outcome (n = 334)**^a^			<0.001
Complete recovery	8 (4.4)	50 (32. 7)	Ref
Improved	126 (69.6)	59 (38.6)	<0.001
Not improved	29 (16.0)	19 (12.4)	<0.001
Death	18 (9.9)	25 (16.3)	0.002
**Discharge diagnosis (n = 252)**			<0.001
Tuberculosis	2 (2.0)	1 (0.7)	0.653
HIV and opportunistic infections	14 (13.7)	83 (55.3)	<0.001
Lower Respiratory Tract Infection	18 (17.6)	20 (13.3)	0.811
Upper Respiratory Tract Infection	2 (2.0)	1 (0.7)	0.653
Fever	6 (5.9)	8 (5.3)	0.749
Septicemia	17 (16.7)	15 (10.0)	Ref
Other	43 (42.2)	22 (14.7)	0.268

Chi-squared or Fisher exact testing

a. Outcome data was derived from several variables in the surveillance database, including “outcome”, “discharge status” and “discharge type”. If the patient was discharged with consent the patient was classified as improved; if discharge was against advice, the patient was recorded as not improved.

**Fig 3:**
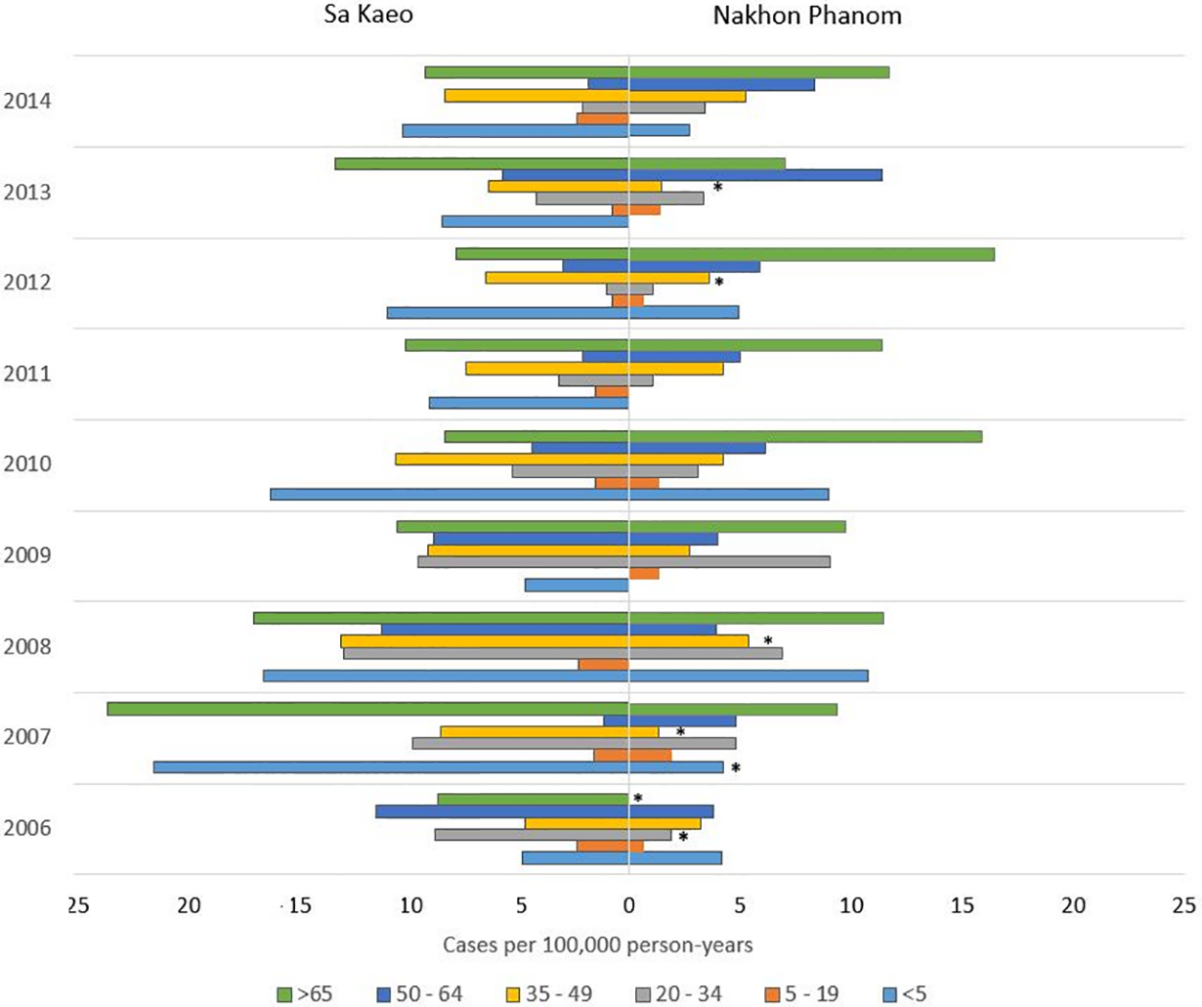
**Incidence of invasive non-typhoidal Salmonellosis by age group and year in Sa Kaeo (SK, left) and Nakhon Phanom (NP, right) provinces, 2006–2014.** RR = relative risk, measured differences between SK and NP. Relative risk is given with upper and lower 95% confidence intervals. * designates age group differences between NP and SK within the specified year (p-value < 0.05) and likely to be driving the differences.

The incidence of iNTS was 4.0 cases/100,000 person-years (95% CI 3.5–4.5) in NP and 6.4 cases/100,000 person-years (95% CI 5.7–7.1) in SK (p < 0.0001). At both sites the highest incidence of iNTS was seen in adults > 65 years of age (NP: 10.3, 95% CI = 7.4–13.1; and SK: 11.9, 95% CI = 8.7–15.1; Fig 3). However, in SK high incidence was also seen in those < 5 years of age and in the 35–49 year old group (8.2 and 11.3 cases/100,000 person-years respectively). This was statistically significant when compared to NP (p = 0.0003 and p < 0.0001 respectively) where overall incidence was calculated at 4.1 and 3.9 cases/100,000 person-years.

**Fig 4:**
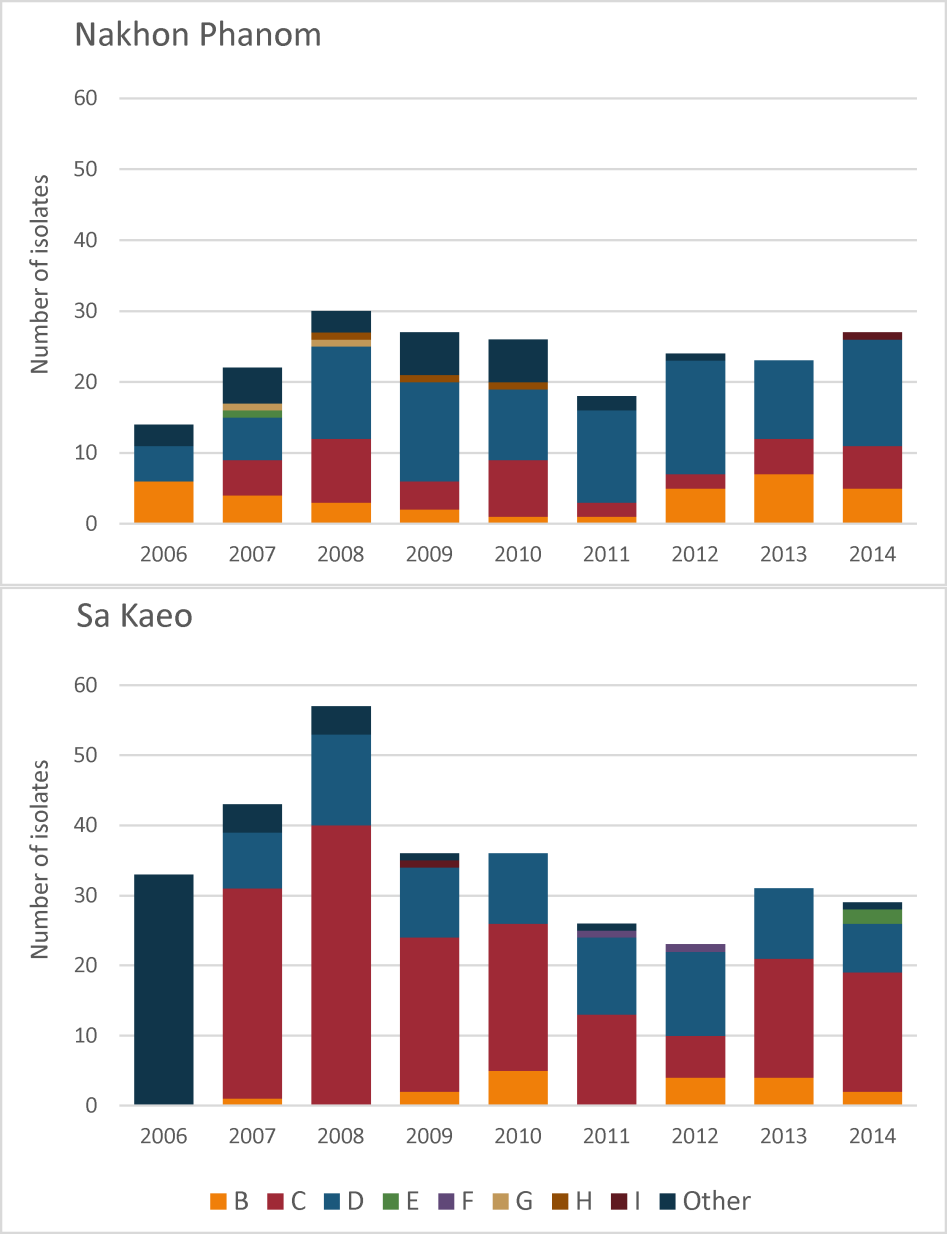
**Distribution of non-typhoidal *Salmonella* serogroups causing invasive infection by year in Nakhon Phanom (top) and Sa Kaeo (bottom), 2006–2014**. “Other” includes non-typed *Salmonella enterica* isolates and isolates from serogroups with few isolates.

The majority of isolates, 308/525 (58.7%), were typed to the serovar level using both O and H antigens; the rest were typed to serogroup level using O antigen sera only (Table 2). Serogroups B, C, and D were the most common in both provinces (443/525; 84.4%), with distinct geographic and temporal trends (Fig 4). In NP, serogroup D was the most commonly isolated throughout the study period, whereas in SK, this was serogroup C. SK showed an increased proportion of serogroup D isolates during 2011–2014 (32.3–52.2%) compared to 2007–2010 (18.6–27.8%). The majority of serogroup D isolates were *Salmonella* serovar Enteritidis in both SK (58/81, 71.6%) and NP (61/103, 59.2%). Serogroup C isolates were found more frequently in SK (166/314, 52.9%) compared to NP (41/211, 19.4%), with *Salmonella* Choleraesuis being the dominant serovar. Of the 52/525 (9.9%) isolates in serogroup B, 21 were designated as I 4,[5],12:i:-, these are monophasic variants and were recorded as likely *Salmonella* Typhimurium isolates (9 isolations from SK during 2009–2012 and 12 from NP during 2012–2014). A total of 28/525 isolates (5.3%) were not characterized beyond their biochemical profile and are recorded as *Salmonella enterica* (Table 2).

**Table 2:** Distribution of invasive non-typhoidal Salmonella serovars isolated in two rural Thai provinces between 2006 and 2014.

O Group	Serovar/Serogroup	Total n = 525	Nakhon Phanom n = 211	Sa Kaeo n = 314
B	Group B^a^ (no serovar data)	25 (4.8)	19 (9.0)	6 (1.9)
B	*Salmonella* serovar Stanley	6 (1.1)	3 (1.4)	3 (1.0)
B	*Salmonella* serovar Typhimurium	21 (4.0)	12 (5.7)	9 (2.9)
C	Group C^a^ (no serovar data)	42 (8.0)	21 (10.0)	21 (6.7)
C	*Salmonella* serovar Albany	3 (0.6)	1 (0.5)	2 (0.6)
C	*Salmonella* serovar Bareilly	2 (0.4)	1 (0.5)	1 (0.3)
C	*Salmonella* serovar Choleraesuis	152 (29.0)	12 (5.7)	140 (44.6)
C	*Salmonella* serovar Javiana	1 (0.2)	1 (0.5)	0
C	*Salmonella* serovar Montevideo	1 (0.2)	1 (0.5)	0
C	*Salmonella* serovar Rissen	6 (1.1)	4 (1.9)	2 (0.6)
D	Group D^a^ (no serovar data)	63 (12.0)	42 (19.9)	21 (6.7)
D	*Salmonella* serovar Djakarta	1 (0.2)	0	1 (0.3)
D	*Salmonella* serovar Enteritidis	119 (22.7)	61 (28.9)	58 (18.5)
D	*Salmonella* serovar Panama	1 (0.2)	0	1 (0.3)
E	Group E^a^ (no serovar data)	3 (0.6)	1 (0.5)	2 (0.6)
F	*Salmonella* serovar Aberdeen	2 (0.4)	0	2 (0.6)
G	Group G^a^ (no serovar data)	2 (0.4)	2 (0.9)	0
H	Group H^a^ (no serovar data)	3 (0.6)	3 (1.4)	0
I	*Salmonella* serovar Hvittingfoss	2 (0.4)	1 (0.5)	1 (0.3)
Other	*Salmonella* other^b^	41 (7.8)	15 (7.1)	26 (8.3)
	*Salmonella* enterica^c^	28 (5.3)	11 (5.2)	17 (5.4)
	*Salmonella* serovar Urbana	1.(0.2)	0	1.(0.3)

^a^ Typing performed using O-group antigen sera only, serovar status not determined

^b^ Multi-group screening using O group antisera showed the isolate not to be in O Group A-I.

^c^. Isolates not typed

**Fig 5:**
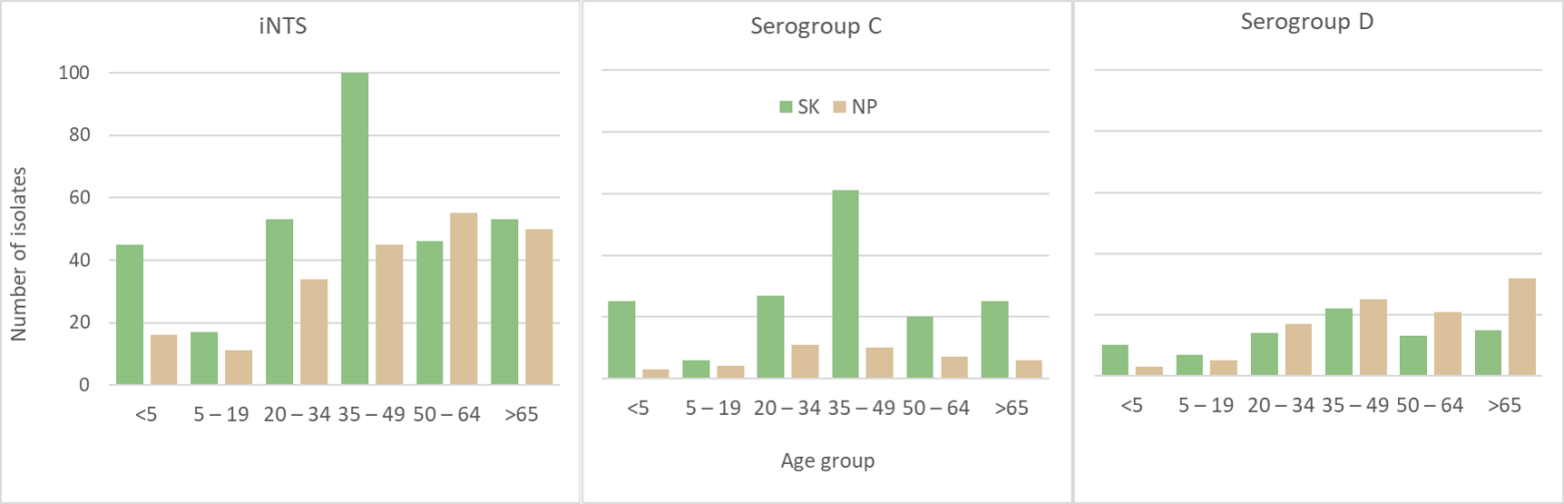
Age distributions for the major serogroups of invasive non-typhoidal *Salmonella* in Nakhon Phanom and Sa Kaeo provinces.

Fig 5 shows the age distributions of the major serogroups by province. Adults in the age bracket 35–49 years have the largest number of serogroup C infections, particularly *S*. Choleraesuis. Children under 5 years of age carried 11.6% of the iNTS infections and 29/61 (47.5%) of these were serogroup C, with 22/29 being *S*. Choleraesuis—all in SK province. For *S*. Enteritidis infections, these mainly affected those >65 years old in NP, while in SK the 35–49 year old age group was most affected.

**Fig 6:**
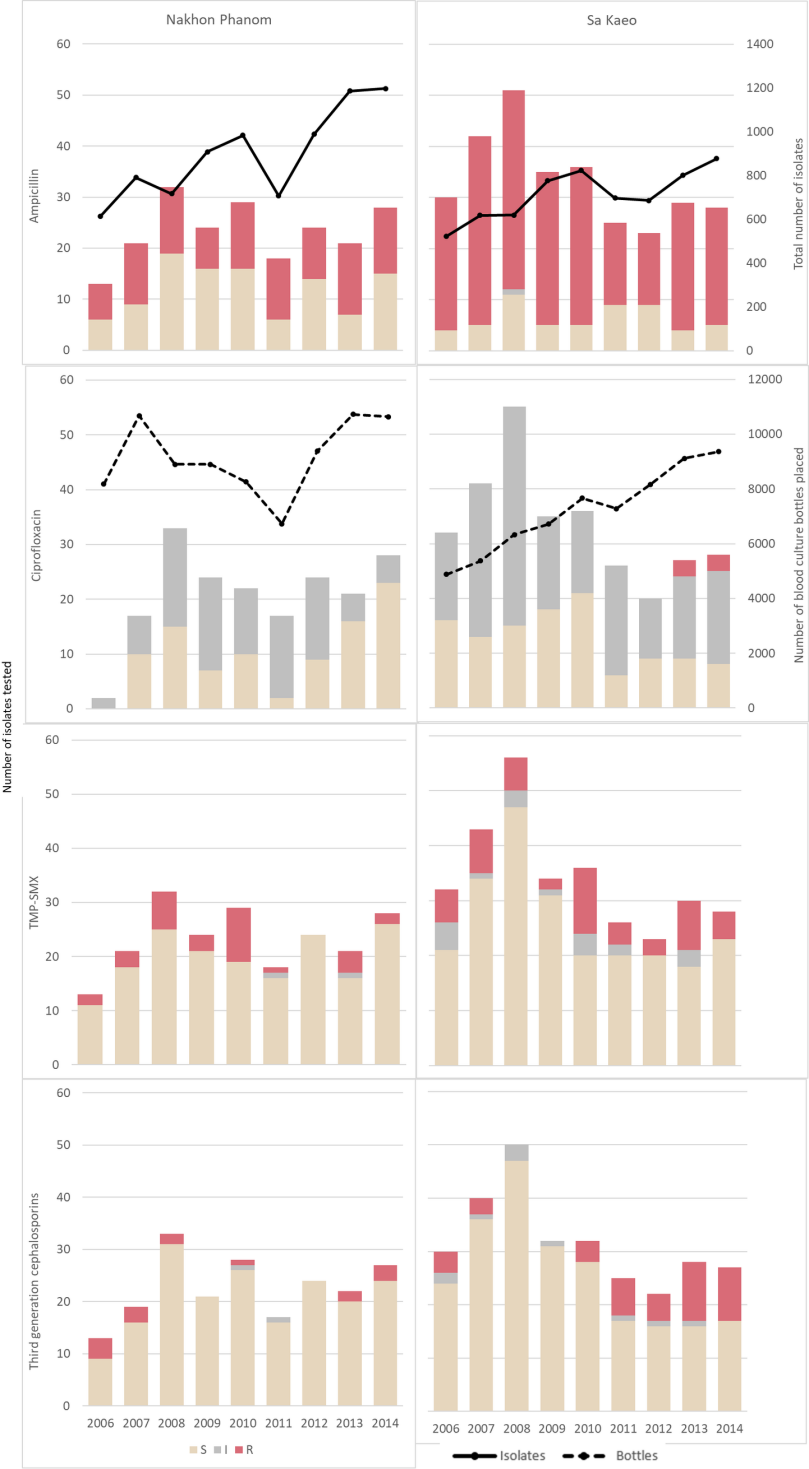
Antibiotic resistance among invasive non-typhoidal *Salmonella* isolates by province and year. Solid line graph shows number of positive blood cultures and the dashed line indicates the total number of bottles incubated by year (z-axis). TMP-SMX = trimethoprim-sulfamethoxazole.

A total of 516/525 (98.3%) isolates were tested for antimicrobial resistance, and of these, 353/516 (68.4%) were resistant to at least one of the antibiotics tested (Table 3). If all non-sensitive results (intermediate and resistant) are considered resistant, this increases to 433/516 (83.9%). Ampicillin resistance was present in 343/503 isolates tested (68.2%), with ampicillin resistance more common in SK (241/299, 80.6%) than NP (102/204, 50.0%; p<0.001). Serogroup C isolates had the highest levels of ampicillin resistance in both provinces (SK: 152/158, 96.2%; NP: 31/40, 77.5%; p<0.001). The resistance profiles for the three remaining antibiotic classes tested: fluoroquinolones (ciprofloxacin), folate pathway inhibitors (TMP/SMX), and third-generation cephalosporins (cefotaxime or ceftazidime) showed >10% antimicrobial resistance among the last two classes at 17.0% (87/512) and 12.2% (59/484), with similar rates of resistance between provinces. Ciprofloxacin (the fluoroquinolone tested), had low rates of resistance (1.2%, 6/482), but high levels of intermediate susceptibility (56.8%, 274/482). Rates of ciprofloxacin intermediate susceptibility were particularly high for serogroups C and D, compared to serogroup B (Table 3). No temporal trends in antibiotic resistance were observed (Fig 6), and no correlation was observed between serogroup and mortality.

**Table 3:** Antimicrobial susceptibility^a^ profiles for non-typhoidal *Salmonella* isolates by serogroup and province.

Serogroup	Antibiotic	Number of Isolates Tested		Nakhon Phanom (NP) n (%)			Sa Kaeo (SK) n (%)		
		NP	SK	S	I	R	S	I	R
B (n = 36)	Ampicillin	34	17	18 (52.9)	0	16 (47.1)	6 (35.3)	0	11 (64.7)
	Ciprofloxacin	28	18	23 (82.1)	5 (17.9)	0	13 (72.2)	5 (27.8)	0
	TMP-SMX^b^	34	18	31 (91.2)	1 (2.9)	2 (5.9)	17 (94.4)	0	1 (5.6)
	3G Cephalosporins^c^	34	17	32 (94.1)	0	2 (5.9)	15 (88.2)	0	2 (11.8)
C (n = 186)	Ampicillin	40	158	9 (22.5)	0	31 (77.5)	5 (3.2)	1 (0.6)	152 (96.2)
	Ciprofloxacin	37	158	17 (45.9)	20 (54.1)	0	54 (34.2)	101 (63.9)	3 (1.9)
	TMP-SMX	39	163	19 (48.7)	1 (2.6)	19 (48.7)	108 (66.3)	14 (8.6)	41 (25.2)
	3G Cephalosporins	34	144	24 (70.6)	2 (5.9)	8 (23.5)	99 (68.8)	8 (5.6)	37 (25.7)
D (n = 150)	Ampicillin	98	78	60 (61.2)	0	38 (38.8)	32 (41.0)	0	46 (59.0)
	Ciprofloxacin	92	76	34 (37.0)	58 (63.0)	0	25 (32.9)	49 (64.5)	2 (2.6)
	TMP-SMX	99	79	93 (93.9)	0	6 (6.1)	73 (92.4)	0	6 (7.6)
	3G Cephalosporins	99	79	99 (100.0)	0	0	79 (100.0)	0	0
All (n = 516)	Ampicillin	204	299	102 (50.0)	0	102 (50.0)	57 (19.1)	1 (0.3)	241 (80.6)
	Ciprofloxacin	182	300	87 (47.8)	95 (52.2)	0	115 (38.3)	179 (59.7)	6 (2.0)
	TMP-SMX	204	308	170 (83.3)	2 (1.0)	32 (15.7)	234 (76.0)	19 (6.2)	55 (17.9)
	3G Cephalosporins	198	286	181 (91.4)	2 (1.0)	15 (7.6)	232 (81.1)	10 (3.5)	44 (15.4)

S = sensitive, I = intermediate, and R = resistant.

^a^ Susceptibility tests were interpreted using the 2017 Clinical and Laboratory Standards Institute (CLSI) breakpoints for disk diffusion, 27^th^ ed. [11]

^b^ TMP-SMX Trimethoprim–Sulfamethoxazole

^c^ 3G (third-generation) cephalosporin data combined from Cefotaxime (n = 504) and Ceftazidime (n = 422) with 93% of results being concordant. Borderline zonal differences (that moved the classification between S/I and I/R) were noted in 13/42 data points; the first classifier was chosen. The remaining 29 discordant results were not included in the analysis

Data collated from both FA and MB bottles.

Multi-drug resistance (MDR) was identified in 99/516 isolates (19.2%), with higher rates in SK (76/310, 24.5%) compared to NP (23/206, 11.2%; p-value < 0.001). The distribution of MDR isolates among the different serogroups was 4/52 (7.7%) in B, 75/204 (36.8%) in C, 6/179 (3.4%) in D (p-value < 0.001) and 1/12 (8.3%) in serogroups E, F, G, H and I combined. Isolates grouped as “Other” showed 9/42 (21.4%) being MDR (including 4/27 (14.8%) *Salmonella enterica* isolates). A single serogroup C isolate (unknown serovar) from SK in 2014 was resistant to all 4 classes of antibiotic tested. Twenty isolates were fully resistant to three classes of antibiotics (17 serogroup C with 14 being *S*. Choleraesuis, and one each in serogroup B, D and E) and of those, 13/20 (65.0%) showed intermediate susceptibility to the fourth class of antibiotic (12 being Ciprofloxacin).

Examination of the 204 serogroup C isolates with AST data showed 164 (80.4%) were from SK with 140/164 being *S*. Choleraesuis (85.4%) and the remaining 24/164 (14.6%) other serogroup C isolates. For *S*. Choleraesuis 43/140 (30.7%) isolates were MDR (all made before 2013), while other serogroup C isolates had higher MDR rates (15/24 (62.5%)), with isolations made in 2013 and 2014. In NP only 30% of the serogroup C isolates were *S*. Choleraesuis (12/40) with 7/12 being MDR (58.3%); 4 isolated in 2010 and the remainder in 2013–2014. For the other serogroup C isolates in NP 10/28 (35.7%) were MDR, all isolated before 2011. No *S*. Enteritidis isolates were MDR, whereas 6/65 (9.2%) of other serogroup D isolates were.

Conversely, 82/516 isolates (15.9%) were fully susceptible to all antibiotics tested. An additional 78 had intermediate sensitivity to ciprofloxacin only, and 2 had intermediate susceptibility for two of the tested antibiotics. Resistance to ampicillin alone (mono-resistance) was the most commonly observed resistance pattern (212/503, 42.1%). Of these 101/212(47.6%) had intermediate susceptibility to ciprofloxacin and 3/212 (1.4%) had intermediate susceptibility to TMP/SMX. All were susceptible to third-generation cephalosporins.

Discharge diagnosis was available for 252/525 (48.0%) iNTS case-patients (Table 1). The most common diagnosis was HIV and opportunistic infections in 97/252 patients (38.5%), and was more often seen in SK (83/97) than NP (14/97) (p-value <0.001). The clinical outcomes for case-patients with diagnoses of HIV or opportunistic infections were known for 91/97 (Table 4): 20/97 (20.6%) died. Five of 16 deaths in SK (31.3%) were from patients with MDR iNTS. The discharge diagnosis of septicemia was reported for only 32/252 case-patients (12.7%) with *Salmonella* being specifically mentioned in 20 of the 32 diagnoses. Death occurred in 2/32 case-patients, and 5 did not improve (3 were transferred to another hospital and 2 were discharged against medical advice). None of these 7 case-patients were MDR. Improvement or complete recovery was noted in 24/32 (75.0%) septicemia case-patients, 6 of which were MDR.

**Table 4:** Association of discharge diagnosis and serogroup with case outcome among patients with invasive non-typhoidal Salmonellosis.

Discharge diagnosis (n = 243)	Outcome (n = 334)							
	Death (n = 43)		Not Improved (n = 48)		Improved (n = 185)		Complete Recovery (n = 58)	
	No. Cases	No. MDR^a^	No. Cases	No. MDR	No. Cases	No. MDR	No. Cases	No. MDR
HIV and opportunistic infections	20	10	11	2	39	10	21	5
Respiratory Tract Infection	4	2	6		19	4	11	3
Other	4		12	2	24	4	10	1
Fever	3		1		12	5	2	
Septicemia	2		5		19	5	5	1
Tuberculosis					2	1	1	
**Serogroup (n = 334)**								
B	3	1	9	2	20	2	5	1
C	10	1	16	1	55	13	2	6
D	20	4	21	2	78	12	35	1
Other	10	2	2		32	10	11	2

a. MDR = multidrug resistant

Data on clinical outcomes were available for 334/525 cases (63.6%, Table 1). Forty-three patients (12.9%) died and a discharge diagnosis was available for 33 (Table 4) showing that 20/33 (60.6%) patients were HIV positive. The median age of patients who died was 46 years old with no deaths in children <5; 1/43 (2.3%) 5–19 years who was HIV positive; 8 (18.6%) in 20–34 years of which 6 (75.0%) were HIV positive; 16 (37.2%) in the 35–49 age group with 10 (62.5%) being HIV positive; 10 (23.3%) for adults aged 50–64 years of whom 30% were HIV positive; and 7 (16.3%) in those > 65 years, none of whom were HIV positive. The majority of patients had a complete recovery or showed improvement and were discharged from the hospital (243/334 (72.6%)). No improvement was seen in 48/334 patients (14.4%): 20 patients were discharged against medical advice, and 28 patients were transferred to another hospital. There were statistically no differences when comparing serogroups. Of the 329 case-patients with AST and outcome data 41/329 (12.5%) died with eight (19.5%) isolates being MDR. There was no statistically significant difference between outcomes for case-patients with MDR and pan-sensitive isolates.

## Discussion

Our population-based surveillance in rural Thailand over the past 9 years showed *Salmonella* to be the fifth leading cause of bloodstream infections [15], and this study is a detailed, retrospective exploration of the epidemiology and antimicrobial susceptibility patterns of the iNTS patients and isolates. Annual incidence rates for iNTS hospitalizations over the study period ranged from 2.2 to 10.7 cases/100,000 person-years in the two provinces. These are the first population-based estimates of iNTS from Southeast Asia, and should be considered as minimal estimates of the burden in Thailand. These numbers are higher than the incidence rates of high income Western countries where the overall approximate annual incidence of 1/100,000 population was calculated from data collected between 2000 and 2007 [16]. They are comparable with estimates from southern Africa at 1.6/100,000 and well below documented incidence rates in eastern and western Africa at 164 and >600/100,000 respectively [2]. Although other incidence data from SE Asia are not available for comparison, the proportion of NTS among bloodstream infections (4.9% SK and 2.6% NP) was similar to rates found in urban environments in Thailand. Kiratisin [17] in a 2005 study of ambulatory and hospitalized patients at a large, 2400 bed, university hospital in Bangkok, found 3.8% of blood culture isolates were NTS. In neighboring countries such as Laos and Vietnam typhoidal *Salmonella* predominated until recently. The most common community-acquired bacteremia identified in Vientiane from 2000–2004 was *Salmonella enterica* serovar Typhi, accounting for 50.9% of cases [18]. In southern Vietnam from 2005–2008 4.5–7.1% of all isolates from blood were *Salmonella* Typhi, whereas 2.7–5.7% were iNTS [19].

The most common serovars causing bacteremia in our study were *S*. Choleraesuis and S. Enteritidis covering 51.6% of all iNTS isolated over the 9 year period. These serovars belong to serogroup C and D respectively, which accounted for 74.5% of all isolates in our study. Serogroup C predominated in SK until 2010 when the serogroup distribution changed, through a decrease in the number of serogroup C cases rather than an increase in serogroup D cases. This pattern is mirrored in the HIV-infected subset of patients, with 76.8% and 78.6% of the iNTS infections in SK and NP respectively. Sirichote et al. [20] demonstrated these two serovars were more commonly isolated from blood than stool in central Thailand, which differs from Africa where *S*. Enteritidis and *S*. Typhimurium are the serovars causing the majority of iNTS disease [2, 3, 21].

Thailand’s declining HIV prevalence has been attributed to successful HIV prevention programs that were implemented between 1990 and 2010 [22]. It is possible that higher iNTS incidence rates in SK during this period may have been due to opportunistic infections, and once antiretroviral treatments to reverse immunosuppression were implemented, iNTS incidence rates were similar to NP, with fewer cases in HIV-positive patients. Reddy et al. [23] reported a strong positive correlation of iNTS with HIV, and a negative correlation of HIV with *S*. Typhi in 7 African countries, with similar reports from several Asian countries including Thailand [24, 25]. A link between NTS bacteremia and HIV infection has been reported in several studies in Thailand [26, 27]. In a study based in lower part of northeast Thailand, Ubon Ratchatani, from 1989–1998, Chierakul et al. [24] noted a rise in serogroup D *Salmonella* bloodstream infections that coincided with the rise in HIV prevalence. Some 20 years later, we still see serogroup D isolates predominating in northeast Thailand, however in SK in eastern Thailand, with statistically higher numbers of iNTS HIV positive cases until 2010, serogroup C iNTS predominated. Kiratisin [17] also noted an increase of *Salmonella* group C isolates causing bacteremia particularly among HIV-infected patients in a study focused in Bangkok, of note was their high rate of MDR isolates. The higher burden of iNTS in Sa Kaeo may be associated with the higher prevalence of HIV in this province [28].

The high levels of antimicrobial resistance is cause for concern, especially in patients with underlying conditions, as the presence of resistance could further complicate patient management. Antimicrobial resistance was commonly found for ampicillin (68.2%), TMP/SMX (17.0%), ciprofloxacin (1.2%) and third-generation cephalosporins (12.2%). These are similar to rates found in a tertiary-care setting in Bangkok among children, where rates were 68.3%, 33.9%, 3% and 17.4% respectively [29]. For ciprofloxacin our intermediate resistance was at 56.8%, this is important to note as the drug of choice by Thai physicians for non-invasive/uncomplicated salmonellosis are the fluoroquinolone derivatives like ciprofloxacin (S. Thamthitiwat personal communication) and rapid decreases in fluoroquinolone susceptibility seen among iNTS isolates in other Asian settings. A 2009 multi-national study showed a high prevalence of reduced susceptibility to ciprofloxacin among non-typhoidal *Salmonella* strains from Taiwan (48.1%), Thailand (46.2%), Korea (36.5%), Singapore (24.5%), Philippines (14.9%), Hong Kong (7.1%), and Sri Lanka (8.0%) [30]. The increasing trend of third-generation cephalosporin resistance, most noticeably in SK, is worrying because it is considered a drug of choice for community-acquired invasive bacterial infection in Thailand. High levels of resistance to third-generation cephalosporins and fluoroquinolones in NTS isolates have been reported in Taiwan and Thailand [20, 30]. Information on iNTS resistance is limited. Kulwichit et al. [31] noted that enrofloxacin, a veterinary fluoroquinolone, is used extensively in the poultry, swine, and seafood industries in Thailand, and ceftiofur, a third-generation cephalosporin, in swine production. Trying to determine whether such antimicrobial resistance is linked to food consumption and/or the excessive use of antimicrobials in animal husbandry would be helpful to guide antibiotic stewardship efforts.

Over 70% of patients recovered after their iNTS infection and 12.9% of patients died. The case fatality rates of HIV-infected and non-infected individuals, and MDR and non-MDR patients were not significantly different which differs than what has been seen in other settings [4]. Our documented case fatality rate is below that estimated by Ao et al. globally among iNTS patients at 20% [7], and the 26% calculated for iNTS in Vietnam [25]. Our case fatality rate is also substantially below that previously documented in Thailand [32] which approached 60% in HIV-infected patients. There are several possible explanations for these discrepancies: our study only captured hospital deaths and it has been shown that in rural Thailand many deaths occur following hospital discharge [33]; this could also reflect differences in patient populations (different age distributions, fewer young children, less co-morbid conditions).

The age distribution of patients with iNTS in SK show a distinct bimodal distribution for most serotypes with peaks occurring in children around 1 year of age and in adults of 35–49 years. Many more infant infections are seen in SK compared to NP and we believe these differences to be real and not a reflection of surveillance or laboratory practice differences between sites. The SK case distribution by age group is similar to that seen in high incidence HIV populations in Africa [7]. An explanation of this difference is not obvious to the authors.

Little seasonality was seen to our iNTS isolates, with no increase during the rainy season. Several studies from Africa have indicated a seasonality to iNTS infections, and these often coincided with the seasonal increase in malaria [34–36]. The rural agrarian communities of these two provinces may indicate that exposure is through infected animal or animal products. *S*. Choleraesuis causes illness in pigs, and infects humans through contact with contaminated animals or environments, and is especially noted in immunocompromised individuals [37]. *S*. Enteritidis is mainly associated with poultry and eggs [38, 39].

There are several limitations to this study. Our study likely underestimates the overall incidence of iNTS in these rural provinces: we did not capture non-hospitalized iNTS cases; pathogen isolation was likely limited by patients having received antibiotics before arrival to the hospital [40]; and blood culture was performed at physician discretion which may have resulted in underestimation of cases through underutilization of this service, or bias in culturing practice based on symptom presentation. The low sensitivity of blood culture in cases of *Salmonella* infection [41] also results in a likely underestimate of cases. We did not collect nationality data, so it is likely that Cambodians and Laotian cases were included which would result in a slight overestimation of incidence. Both demographic and clinical characteristics of patients were only available for a subset of cases, which limited our ability to identify risk factors. Because outcome data only covered the period of hospital admission, it is likely that the case fatality rate was underestimated. Kanoksil showed that in rural Thailand many of the bacteremic patient deaths occurred following hospital discharge [33]. Laboratory methods for the typing of *Salmonella* isolates changed over time, so serotype information was inconsistent and limited.

Despite these limitations, we demonstrate the value of population-based surveillance to describe iNTS disease burden, filling a knowledge gap in Southeast Asia. AMR is widely recognized as a major threat to global health security and monitoring and reporting of AMR surveillance data at national and regional levels enables physicians to guide local empirical therapy, and for countries to develop and implement timely and adequate countermeasures. This data is essential at the global level to understand antimicrobial resistance patterns and to monitor progress in responding to increasing resistance so the spread of MDR bacteria does not reach the point where prevention, control and treatment are severely compromised. Our data should provide guidance on prevention strategies including initiatives to develop NTS vaccines [42]. In Africa, the paucity of serovars makes vaccination attractive, however, our studies shows a lower burden of disease with a larger diversity of types, making alternate prevention measures more attractive. This study fills several significant gaps in our knowledge of iNTS in Thailand and Southeast Asia. Continued and expanded surveillance is needed to monitor the rising levels of antimicrobial resistance, expand on the risk factors, patient outcomes, and exposures associated with iNTS in Thailand and Southeast Asia.

## Supporting information

S1 Checklist STROBE checklist. Completed STROBE checklist for cross-sectional studies completed by corresponding author.
(DOC)

S1 Text Author responses letter. (DOCX)

S1 Data Supplemental data. (PDF)
