# Supplementary material for: Epidemiology and antimicrobial resistance of invasive non-typhoidal Salmonellosis in rural Thailand from 2006-2014
Source: PLoS Negl Trop Dis. 2018 Aug 6;12(8):e0006718. doi: 10.1371/journal.pntd.0006718 (PMC6095622; doi:10.1371/journal.pntd.0006718)
Supplement: S1 Checklist — Completed STROBE checklist for cross-sectional studies completed by corresponding author. (DOC) [file pntd.0006718.s001.doc]

STROBE Statement—Checklist of items that should be included in reports of ***cross-sectional studies***

|  | Item No | Recommendation |
| --- | --- | --- |
| **Title and abstract** | 1 | Title page, page 1 line 1 |
|  |  | Abstract. Page 2, lines 24-44 |
| Introduction | | |
| Background/rationale | 2 | Introduction, paragraph 1 |
| Objectives | 3 | Introduction, paragraph 2, second sentence |
| Methods | | |
| Study design | 4 | Methods, second section (Study setting and population), paragraph 1 |
| Setting | 5 | Methods, second section (Study setting and population), paragraph 1 |
| Participants | 6 | Methods, second section (Study setting and population), paragraph 1 |
| Variables | 7 | Specimen collection and laboratory testing – paragraph 4 Antimicrobial sensitivity testing (AST)  Specimen collection and laboratory testing – paragraph 5 Salmonella typing  Clinical and demographic variables – Table 1 |
| Data sources/ measurement | 8* | AST – laboratory data CLSI guidelines (paragraph 4, starting line 123)  Typing – laboratory data (paragraph 5, starting line 130) |
| Bias | 9 | Line 140 |
| Study size | 10 | Not applicable, observational study |
| Quantitative variables | 11 | Methods, CLSI guidelines for AST data reference 11 |
| Statistical methods | 12 | (*a*) Methods, Statistical data analysis (paragraph 2 and 3) |
|  |  | (*b*) Not applicable |
|  |  | (*c*) Not applicable |
|  |  | (*d*) Not applicable |
|  |  | (*e*) Not applicable |
| Results | | |
| Participants | 13* | (a) Figure 2 |
|  |  | (b) Figure 2 |
|  |  | (c) Figure 2 |
| Descriptive data | 14* | (a) Table 1, results line 166 |
|  |  | (b) Table 1 and Table 4 |
| Outcome data | 15* | Table 1 – clinical outcome and diagnosis. Results paragraphs 10 and 11  Table 2 and Fig 5 – isolate serovar; results paragraphs 4 and 5  Table 3 – AST summary,; results paragraph 6  Incidence – results paragraph 3 |
| Main results | 16 | (*a*) Incidence - Results paragraph 3 |
|  |  | (*b*) Not applicable |
|  |  | (*c*) Not relevant |
| Other analyses | 17 | All covered above. |
| Discussion | | |
| Key results | 18 | Results, paragraph 3 – incidence  Results, paragraph 4 – serovars  Results, paragraph 6 – AST data |
| Limitations | 19 | Discussion, line 327 |
| Interpretation | 20 | Discussion, last paragraph |
| Generalisability | 21 | Discussion, last paragraph (lines 338-350) |
| Other information | | |
| Funding | 22 | Moved to online submission form at editor request |

*Give information separately for exposed and unexposed groups.

**Note:** An Explanation and Elaboration article discusses each checklist item and gives methodological background and published examples of transparent reporting. The STROBE checklist is best used in conjunction with this article (freely available on the Web sites of PLoS Medicine at http://www.plosmedicine.org/, Annals of Internal Medicine at http://www.annals.org/, and Epidemiology at http://www.epidem.com/). Information on the STROBE Initiative is available at www.strobe-statement.org.
