# Supplementary material for: Epidemiology and antimicrobial resistance of invasive non-typhoidal Salmonellosis in rural Thailand from 2006-2014
Source: PLoS Negl Trop Dis. 2018 Aug 6;12(8):e0006718. doi: 10.1371/journal.pntd.0006718 (PMC6095622; doi:10.1371/journal.pntd.0006718)
Supplement: S1 Data — (PDF) [file pntd.0006718.s003.pdf]

Supplemental data to "Epidemiology and antimicrobial resistance of invasive non-typhoidal Salmonellosis in rural Thailand from 2006-2014"

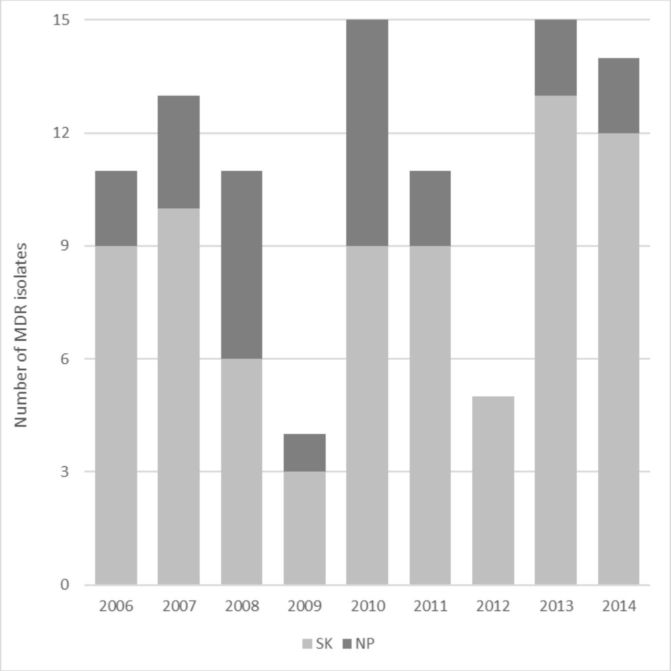

Figure S1: Temporal distribution of multidrug resistant invasive nontyphoidal *Salmonella* isolates by Thai province, 2006-2014.

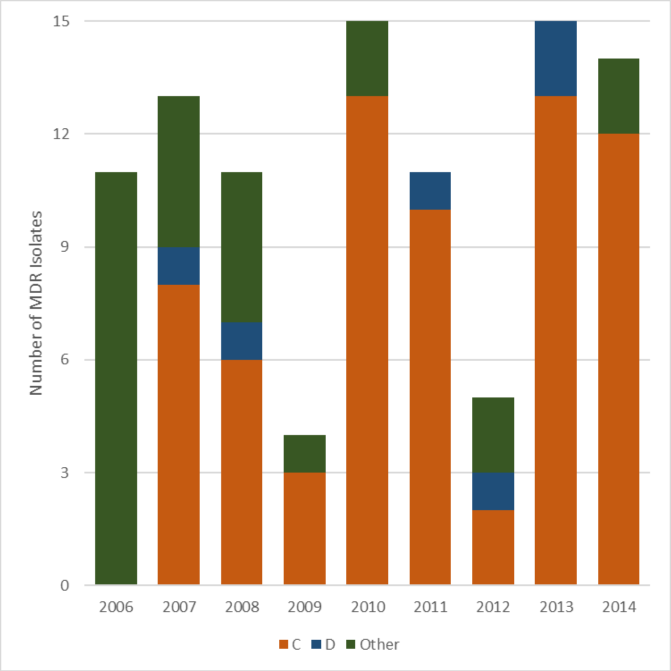

Figure S2: Distribution of multidrug resistant invasive nontyphoidal *Salmonella* isolates by serogroup from 2006 through 2014.

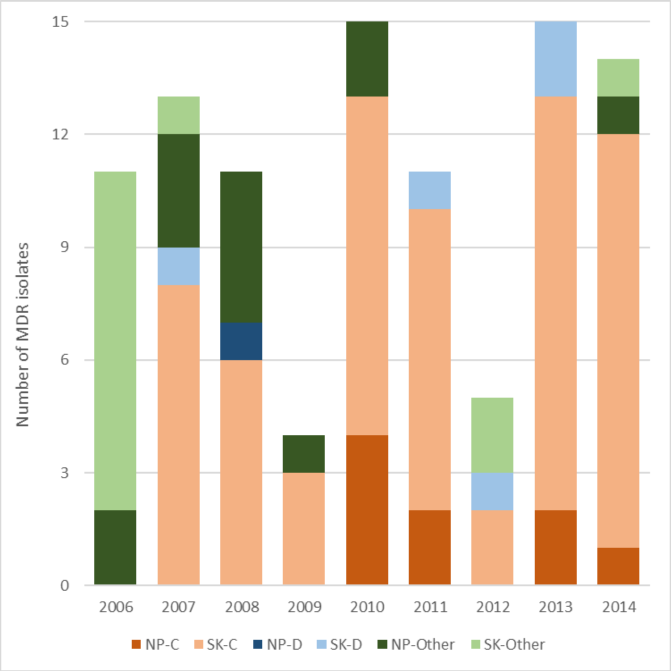

Figure S3: Temporal distribution of multidrug resistant invasive nontyphoidal *Salmonella* isolates by province and serogroup, 2006-2014.
