# Supplementary material for: Epidemiology and antimicrobial resistance of invasive non-typhoidal Salmonellosis in rural Thailand from 2006-2014
Source: PLoS Negl Trop Dis. 2018 Aug 6;12(8):e0006718. doi: 10.1371/journal.pntd.0006718 (PMC6095622; doi:10.1371/journal.pntd.0006718)
Supplement: S1 Text — (DOCX) [file pntd.0006718.s002.docx]

Reviewer's Responses to Questions: PNTD-D-18-00727

**Key Review Criteria Required for Acceptance?**

As you describe the new analyses required for acceptance, please consider the following:

**Methods**

-Are the objectives of the study clearly articulated with a clear testable hypothesis stated?

-Is the study design appropriate to address the stated objectives?

-Is the population clearly described and appropriate for the hypothesis being tested?

-Is the sample size sufficient to ensure adequate power to address the hypothesis being tested?

-Were correct statistical analysis used to support conclusions?

-Are there concerns about ethical or regulatory requirements being met?

Reviewer #1: Are there are no major concerns regarding the methodologies used in this ms. The manuscript clearly describes the methodologies used for the laboratory and the statistical analysis. Could the authors please state be statistical software package used to perform the statistical analysis?

Statement added line 158: All statistical analyses were performed using Stata version 11 (StataCorp, College Station, TX, USA).

Reviewer #2: Objectives of the study were clearly defined as to better understand the burden and antimicrobial resistance of iNTS disease in Thailand.

1. The study was described as a retrospective analysis of NTS blood stream infections. Since the study was approved and conducted as a part of public health surveillance, please briefly describe the normal practice of public health surveillance in Thailand for patients with suspected blood stream infections e.g. clinical indication for blood culture investigation, routine laboratory investigation e.g. culture and identification, serogroup/serovar testing, AST, isolate archive.

Additions have been made to the methodology: Case enrollment (lines 101-105): “Cases were enrolled using the Thai Sepsis Guidelines (2006; updated 2010) based on Systemic Inflammatory Response Syndrome (SIRS). Sepsis is defined as having suspected or confirmed infection with at least two of the following SIRS criteria: temperature >38°C or <36°C, heart rate > 90 beats/min, respiratory rate >20/min or PaCO2 <32 mmHg, white blood cell count >12,000 or <4,000/mm3 or having band form neutrophils >10%.”

Isolate archive was added in line 148. “Glycerol stocks were prepared for -70˚C long-term storage of all isolates.”

The routine investigations have previously been covered in this section, we feel no further elaboration was needed.

2. Please provide a rational on how the 2 sites (SK and NP) were selected. How the 2 sites in rural Thailand can represent the iNTS disease burden in Thailand as in the objective?

Addition made to methods, line 92: “Initially sites were selected for their ability to capture all potential admissions in the provinces, as there are no private or other acute care hospitals in either province.”

3. It is interesting to learn about quality control of the data during the 9 year surveillance with blood culture, sample transfer, microbiological identification of NTS, serogroup/serovar testing, antimicrobial susceptibility testing etc. as a public health surveillance.

Extensive quality checks are implemented throughout all surveillance activities. These include:

- Clearly defined data elements, data review of both collection and entry, system-based controls in the computerized data management system (added lines 151-152).
- Standard quality control procedures in the microbiology laboratory including weekly media checks with control strains, participation in national and external quality assurance programs, confirmatory testing for identification and serogrouping by the Thai NIH on about 10% of isolates. This was detailed in the methods section (paragraph starting at line 113) so not further information was added.

4. In the method referred to using the 2015 CLSI guideline for interpretation of disk diffusion method. Was the AST performed on the archived NTS isolates by the researchers using 2015 interpretative criteria or the AST data was obtained from retrospective hospital lab record review (as S, I, R) for analysis?

This is an important point and we have re-worded this section to clarify exactly how this was tackled. AST testing was performed throughout the study period, and for this manuscript zone diameters were interpreted using the 2015 CLSI guidelines.

Changes: Page 5, lines 132-135: Antimicrobial susceptibility testing (AST) was performed using the disk diffusion method “throughout the study period. Provincial authorities, taking into account local considerations such as available antibiotics and observed resistance patterns, guided antibiotics used for testing. Zone information for this manuscript was interpreted” according to Clinical and Laboratory Standards Institute (CLSI) guidelines 2015 [11].

My concern is on the technique and interpretation of disk diffusion test over a span of 9 year study period in 2 provinces that CLSI guideline has been updated over time and how the authors think the 9 year AST data is comparable.

I hope that the above explanation allays your concerns. We are aware of these issues so were able to deal with this in the manuscript as zone diameters had been recorded.

5. Was the study reviewed and approved by local IRB in Thailand? Please clarify in ethical statement, as it was not mentioned.

Added this statement to the text (line 83-84): “Surveillance is considered a core function of the Thai Ministry of Public Health and as such was determined not to require ethical committee or institutional review board evaluation.”

--------------------

**Results**

-Does the analysis presented match the analysis plan?

-Are the results clearly and completely presented?

-Are the figures (Tables, Images) of sufficient quality for clarity?

Reviewer #1: The results are well presented.

Reviewer #2: The analysis presented matched the analysis plan, results and figures are clearly presented.

1. The yield of blood culture positive for clinically significant pathogens are pretty low (9%). Was there any improvement of the yield rate over time? Was the criteria for blood culture request of those 2 hospitals too broad or due to prior antibiotic use etc.? Please comment.

This use of antibiotics prior to arrival at the hospital is mentioned in the discussion under study limitations (line 344).

2. It will be interesting to present the antibiotic resistance trend of the 2 predominate serovars, enteritidis and choleraesuis or by serogroup with MDR proportion. Suggest results in Line 206-220 may be easier to follow in graph.

We have tried several ways of presenting this data graphically but it requires several different graphs – and we were not able to effectively combine the data types. For this reason we have left the data as is. We have added a supplemental file with additional temporal distributions on the MDR data.

3. Is mortality correlated to certain specific serovars of iNTS?

Sentence added to the results, line 220: No temporal trends in antibiotic resistance were observed (Fig 6), “and no correlation was observed between serogroup and mortality.”

**Conclusions**

-Are the conclusions supported by the data presented?

-Are the limitations of analysis clearly described?

-Do the authors discuss how these data can be helpful to advance our understanding of the topic under study?

-Is public health relevance addressed?

Reviewer #1: The authors I have presented the context of the results quite well in the discussion section. There is a section describing the limitations of the study and these limitations are valid. I'm slightly concerned about the statement in line 252 that "These are the first population-based estimates of iNTS from Southeast Asia" you as firstly this is a hospital-based study and the authors refer to at least two other studies from Vietnam relating to iNTS. The authors please amend or clarify this statement.

The Vietnamese studies are not population based so incidence is not determined. They present prevalence data and we feel that this is clear in the manuscript (rates are not presented but percentages are given). We stand by the statement that these are the first population-based estimates and have not amended this statement. This is a hospital-based study and is clear in the descriptions of our surveillance. This is discussed in the limitations and we note this is an under-estimate of the disease burden.

Reviewer #2: Conclusions supported data presented. Strength and weaknesses and public health relevance were described.

1. I agree with the comment in the last paragraph of discussion that a lower disease burden is reported although the incidence may be underestimated due to several limitations. The results correlated with the report by Haselbeck et al (Ref #8) and should have been discussed. However, a conclusion that NTS is a common cause of bloodstream infection (when NTS is #5 pathogen identified in an average of 4% of total number of pathogenic isolates and less than 0.5% of total blood culture submitted) seemed to contradict with the low disease burden and should have been toned down a bit.

The statement was deleted from the discussion and conclusions – we agree it was a little over the top (line 364). Adjustments were also made in the abstract conclusions. It is not clear to us as to what should be discussed with reference to the Haselbeck paper. At >4500 words we are reticent to add to the manuscript.

2. Changing of laboratory procedures and guidelines e.g. for AST, serotyping etc. made quality control of data challenging. How the authors overcame those challenges should be interesting to the readers who conduct public health surveillance and should be discussed under limitation of the study.

This would be a whole new manuscript.

Laboratory quality standards are discussed in the methods section and data monitoring was added at the request of reviewer #2 (this document page 1 bullet 3).

3. Any comments on the different epidemiology of iNTS in the two provinces e.g. agriculture, animal exposure etc. The HIV status has been discussed on its potential effect to the declined iNTS incidence in SK. For NK, it seems like the incidence did not change. Can you please comment?

The epidemiological differences we understand between the two provinces are highlighted in the paper and we believe stem from the different HIV rates. The decline in iNTS in SK was attributed to the introduction of HIV treatment (paragraph starting at line 288). It is not clear as to what further comment the reviewer would like us to make.

**Editorial and Data Presentation Modifications?**

Use this section for editorial suggestions as well as relatively minor modifications of existing data that would enhance clarity. If the only modifications needed are minor and/or editorial, you may wish to recommend “Minor Revision” or “Accept”.

Reviewer #1: None

Reviewer #2: Discussed in the other sections.

--------------------

**Summary and General Comments**

Use this section to provide overall comments, discuss strengths/weaknesses of the study, novelty, significance, general execution and scholarship. You may also include additional comments for the author, including concerns about dual publication, research ethics, or publication ethics. If requesting major revision, please articulate the new experiments that are needed.

Reviewer #1: This is a particularly worthwhile study and allows a better understanding of iNTS in Thailand and in some ways can be extrapolated to other areas of Southeast Asia.

It is worthwhile noting that the provinces chosen for this study border Laos and Cambodia and maybe considered to be economically poorer provinces than others throughout Thailand and it may be useful for the authors to comment on this fact. Can the authors please comment on the possibility of patients from Laos and Cambodia being included in this study and what impact their inclusion would have on the results?

We did not collect nationality. It is possible that we included Cambodians and Laotians in the numerator, but not denominator which would lead to slight overestimation of incidence. However, I would argue that low culture sensitivity, high pre-culture antibiotic use, and many other factors FAR outweigh this issue. Virtually all of our incidence estimates are underestimates of the true disease burden. A sentence has been added to the study limitations paragraph (line 347).

Also, can the authors please comment on the possible sources of infection given the context of these provinces being economically poorer with a large rural population presumably involved in agriculture and labouring. I cannot see any reference to the occupations of the participants in the study - similar to the previous reference can be authors please comment on the influence of occupation on iNTS.

Reference is made to these rural populations being agrarian (methods, line 96) and we do postulate in the discussion on exposures (paragraph starting at line 336) and contributions made towards AMR (sentence starting line 319). As we have not specifically collected occupational information, I would not be comfortable commenting beyond what we have included.

Reviewer #2: A big series of data from a population-based surveillance for invasive Non Typhoidal Salmonella (iNTS) in 2 provinces in rural Thailand involving almost 150000 blood culture samples during a 9 year surveillance period has been reported. Incidences, epidemiology has been described as well as serogroup/serovar distribution. iNTS isolates are commonly resistant to ampicillin, TMP-SXT and third generation cephalosporin and with a high proportion of reduced susceptibility to CIP and multidrug resistances.

The strength is big series of data over a span of time with supporting laboratory data. The antimicrobial resistance data which is very important but is lacking in Thailand is also reported. The weakness is on study design to capture cases and calculated a disease incidence and how the 2 rural sites could represent Thailand and made the data relevant to Thailand and the region as mentioned in conclusion.
